# Teriflunomide as a therapeutic means for myelin repair

**DOI:** 10.1186/s12974-022-02686-6

**Published:** 2023-01-07

**Authors:** Peter Göttle, Janos Groh, Laura Reiche, Joel Gruchot, Nicole Rychlik, Luisa Werner, Iria Samper Agrelo, Rainer Akkermann, Annika Zink, Alessandro Prigione, Hans-Peter Hartung, Rudolf Martini, Patrick Küry

**Affiliations:** 1 Department of Neurology, Medical Faculty, Heinrich-Heine-University, Moorenstrasse 5, 40225 Düsseldorf, Germany; 2 Department of General Pediatrics, Neonatology and Pediatric Cardiology, Medical Faculty, Heinrich-Heine-University, Düsseldorf, Germany; 3 Department of Neurology, Section of Developmental Neurobiology, University Hospital, Würzburg, Germany; 4 Brain and Mind Center, University of Sydney, Sydney, Australia; 5 Department of Neurology, Palacky University Olomouc, Olomouc, Czech Republic

**Keywords:** Multiple sclerosis, Teriflunomide, Oligodendrocyte, Remyelination, Neuroregeneration

## Abstract

**Background:**

Promotion of myelin repair in the context of demyelinating diseases such as multiple sclerosis (MS) still represents a clinical unmet need, given that this disease is not only characterized by autoimmune activities but also by impaired regeneration processes. Hence, this relates to replacement of lost oligodendrocytes and myelin sheaths—the primary targets of autoimmune attacks. Endogenous remyelination is mainly mediated via activation and differentiation of resident oligodendroglial precursor cells (OPCs), whereas its efficiency remains limited and declines with disease progression and aging. Teriflunomide has been approved as a first-line treatment for relapsing remitting MS. Beyond its role in acting via inhibition of de novo pyrimidine synthesis leading to a cytostatic effect on proliferating lymphocyte subsets, this study aims to uncover its potential to foster myelin repair.

**Methods:**

Within the cuprizone mediated de-/remyelination model teriflunomide dependent effects on oligodendroglial homeostasis and maturation, related to cellular processes important for myelin repair were analyzed in vivo. Teriflunomide administration was performed either as pulse or continuously and markers specific for oligodendroglial maturation and mitochondrial integrity were examined by means of gene expression and immunohistochemical analyses. In addition, axon myelination was determined using electron microscopy.

**Results:**

Both pulse and constant teriflunomide treatment efficiently boosted myelin repair activities in this model, leading to accelerated generation of oligodendrocytes and restoration of myelin sheaths. Moreover, teriflunomide restored mitochondrial integrity within oligodendroglial cells.

**Conclusions:**

The link between de novo pyrimidine synthesis inhibition, oligodendroglial rescue, and maintenance of mitochondrial homeostasis appears as a key for successful myelin repair and hence for protection of axons from degeneration.

## Background

The adult central nervous system (CNS) exhibits only limited regeneration capacities and impaired repair processes in patients with multiple sclerosis (MS) contribute to neurological disability and diminished quality of life on a long-term scale. While relapsing MS (RMS) is amenable to treatment by means of a number of well-established immunomodulatory drugs, the disease per se remains incurable and develops into progressive stages (PMS) with irreversible functional deficits. In this respect, the primary hallmark is the autoimmune mediated breakdown of myelin sheaths and the subsequent loss of mature oligodendrocytes associated with impaired axonal integrity and neurodegeneration [1]. Beyond immunomodulation the current focus lies on regenerative aspects and their potential for clinical translation. Partial replacement of lost oligodendrocytes and myelin sheaths following demyelination of the adult CNS can occur spontaneously as a result of activation of resident oligodendroglial precursor cells (OPCs) [2, 3]. Nevertheless, successful cell replacement and remyelination of denuded axons remain inefficient and should be supported via therapeutic intervention. In such a scenario an active promotion of axonal remyelination will lead to stabilization, electrical insulation, improved trophic and metabolic support thereby eventually preventing neurodegeneration and restoring axonal function [4, 5]. Hence, identification of substances exerting positive effects on oligodendroglial cell differentiation and boosting the endogenous remyelination capacity are of increasing biomedical interest as it represents an unmet clinical need [6, 7]. In addition to numerous drug screening approaches that have been conducted for this purpose (recently summarized in [8]) drug repurposing represents a promising alternative approach as it is thought to accelerate substance identification in a cost efficient manner [9]. Teriflunomide is an approved first line oral immunomodulatory medication for patients with RMS [10–12] acting via inhibition of pyrimidine biosynthesis in activated lymphocytes by selective and reversible blockade of the mitochondrial enzyme dihydroorotate dehydrogenase (DHODH) [13, 14]. DHODH is an inner mitochondrial membrane protein that catalyzes the oxidation of dihydroorotate to orotate, which is further converted into uridine monophosphate from which all other pyrimidine ribonucleotides arise [15]. Beyond its role as an immune modulator we previously described teriflunomide’s potential role in neuroregeneration as it promoted primary OPC differentiation and internode formation, particularly when applied early and in pulses within myelinating neuron/glia co-cultures [16]. Subsequent studies further revealed an association with zymosterol accumulation and teriflunomide to exert promyelinating effects in demyelinated *Xenopus laevis* and mouse spinal cord [17]. We here provide additional evidence for the potential of teriflunomide as a regenerative compound as it was applied in a cuprizone mediated demyelination model suitable to study cellular and subcellular processes leading to axonal remyelination in vivo [18]. We revealed that oral teriflunomide application substantially promotes oligodendroglial differentiation, fosters myelin sheath restoration and restores mitochondrial integrities within the affected corpus callosum (CC).

## Materials and methods

### Ethics statements for animal experiments

Cuprizone mediated demyelination experiments were approved by the authorities at LANUV (Landesamt für Natur, Umwelt und Verbraucherschutz Nordrhein-Westfalen; Az.: 81-02.04.2019.A203) and were carried out according to ARRIVE guidelines. These experimental procedures are characterized by mild severity grade, and therefore, no interventions to reduce pain, suffering and distress were needed.

### Cuprizone diet and drug-treatment

Eight-week-old female C57BL/6 mice (Janvier Labs, Paris, France) were used and all experiments were performed in the animal facility of the Heinrich-Heine-University (Zentrale Einrichtung für Tierforschung und wissenschaftliche Tierschutzaufgaben; ZETT) under pathogen-free conditions and in accordance with ethical care. Only mice of the same age and with body weight between 17 and 19 g (gr) were included and as additional exclusion criterion > 10% weight loss during the experiments was used. Upon delivery, animals were distributed equally into cages (groups) and were given 1 week as acclimatization period before the initiation of the experiments. Using the software G*Power a required size of maximal *n* = 6 animals per group was predicated (see further below for details). This analysis was also necessary to have animal experiments legally granted by the authorities (The Review Board for the Care of Animal Subjects of the district Government) (LANUV, North-Rhine Westphalia, Germany). For the here presented in vivo investigations we finally used at least *n* = 4 animals per experimental group.

Demyelination was induced by feeding 0.2% (w/w) cuprizone [bis(cyclohexanone)oxaldihydrazone]-containing diet from Envigo (Indianapolis, IN, US Cat# TD.140803) and SSNIFF Spezialdiäten GmbH (Soest, Germany; maintenance diet pellets 10 mm, V1534 implemented with 0.2% cuprizone, Sigma-Aldrich, CAS 370-81-0) for 6 weeks. Thereafter animals were given standard rodent chow (SSNIFF, Cat# V1534).

**Fig. 1:**
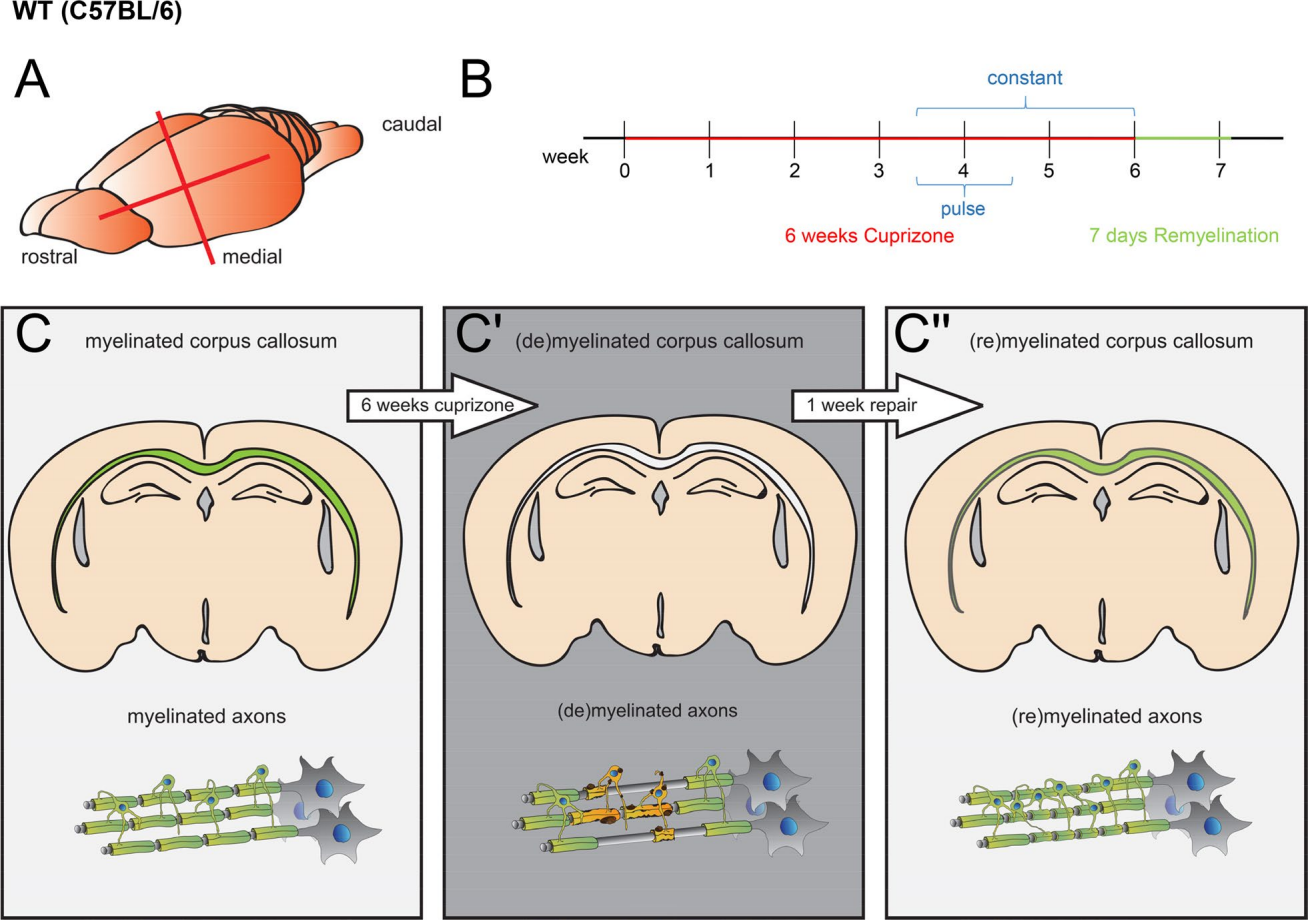
Cuprizone (CPZ) mediated demyelination and teriflunomide treatment. **A** Rostro-caudal directed coronal brain slices were collected between − 1.94 and − 2.34 mm Bregma. **B** Timeline of (**C**–**C**′′) CPZ induced demyelination and of teriflunomide treatment in adult (8 weeks) mice. CPZ treatment lasted for 6 weeks. Teriflunomide was applied orally by means of two different application schemes: (1) early pulse during the fourth week of cuprizone challenge for 7 consecutive days (pulse) or (2) continuously for the final 20 days of cuprizone treatment (constant). Mice were sacrificed after 7 days of remyelination

Teriflunomide (A-771726, Biorbyt, St Louis, US) was dissolved in autoclaved drinking water containing 0.6% Tween 80 at 60 μg per milligram and provided ad libitum. With an approximate consumption of 5 ml per day and 30 gr body weight, this corresponds to a dose of 10 mg/kg body weight per day. This concentration is based on previous animal experiments in other laboratories [19, 20] and comparable to doses used for human patients, when a dose conversion scaling is applied [21]. Non-treated controls received autoclaved drinking water with 0.6% Tween 80 only previously shown to exert no effect on neuroinflammation and neural damage [20]. No daily handling occurred; however, animals (control as well as teriflunomide treated groups) were handled twice a week for weight control. This occurred in parallel (for both groups) and also drinking water was changed twice a week (for both groups). Teriflunomide administration was performed as an early pulse during the fourth week of cuprizone challenge for 7 consecutive days (Fig. 1; pulse) or continuously for the final 18 days of cuprizone treatment (Fig. 1; constant). Following the 6-week demyelination period and teriflunomide treatment (see application scheme in Fig. 1) mice were fed standard rodent chow (SSNIFF, Cat# V1534) for another 7 days to allow newly formed oligodendrocytes (OLs) to remyelinate. Mice were then deeply anesthetized using isoflurane inhalation and transcardially perfused with 20 ml ice-cold PBS followed by 20 ml 4% paraformaldehyde (PFA) in PBS. Brains were then harvested and post-fixation was performed overnight in 4% PFA at 4 °C. Following post-fixation, cryo-protection of mouse brains was performed in 30% sucrose (in PBS) at 4 °C for 24–48 h. Brains were embedded in Tissue-Tek OCT medium (Sakura Finetek Europe, Netherlands), frozen, and stored at − 80 °C until sectioning with a cryostat (Leica CM3050S, Wetzlar, Germany). Coronal 10 µm sections were prepared and stored at − 80 °C. As region of interest the caudal part of the corpus callosum (Bregma − 1.94 to − 2.34 mm) was assessed according to mouse brain atlas (Franklin and Paxinos, 2008).

### Immunohistochemical staining

For immunohistochemical staining, sections were thawed and air-dried for at least 10 min at room temperature (RT). Rehydration was performed for 5 min in distilled water, followed by post fixation with 4% PFA for 10 min. Thereafter sections were transferred to 50% acetone for 2 min at RT, followed by 100% acetone for 2 min at RT, 50% acetone for 2 min and then washed 3 × with Tris-Buffered Saline supplemented with 0.02% Triton X-100; pH 7.6 (TBS-T) for 2 min each. Non-specific staining was blocked with 10% normal goat serum (NGS) (Biozol vector Cat# S-1000, Eching, Germany), 10% normal horse serum (NHS) (Biozol vector Cat# S-2000, Eching, Germany) or 10% normal donkey serum (NDS) (Sigma‐Aldrich Cat# D9663, Taufkirchen, Germany) supplemented with 3% biotin-free bovine serum albumin [BSA (Carl Roth # 0163, Karlsruhe, Germany) in TBS w/o TX-100 for 30–60 min at RT, followed by primary antibody solution application and incubation overnight at 4 °C. The following primary antibodies were used: mouse anti-APC (CC1, 1:300, Sigma-Aldrich, Cat# OP80, RRID:AB_ 2057371); rabbit anti-sex determining region Y-Box 10 (Sox10; 1:100, DCS Immunoline, Hamburg, Germany Cat# S1058C002, RRID: AB_2313583) and goat anti-Sox10 (1:200, R&D System, Minneapolis, US Cat# AF2864; RRID: AB_442208); rat anti-proteolipid protein (PLP, 1:250, kind gift from B. Trapp and R. Dutta, Dept. of Neurosciences, Cleveland Clinic, OH, [22]), mouse anti-mammalian Achaete scute homolog-1 (Mash1, 1:200; [23, 24]) and rabbit anti-mitochondrial outer membrane marker (Tom20; Santa Cruz, Heidelberg, Germany Cat# sc-11415 (FL-145), RRID: AB_2207533). Slices were then washed twice in 1 × TBS for 5 min each and secondary antibodies were diluted 1:200 and applied for 30 min along with 4′,6-diamidino-2-phenylindol (DAPI, 1:50) in PBS. The following secondary antibodies were used: goat anti-rabbit Alexa Fluor 594 (1:200, Thermo Fisher Scientific Cat# A-11037, RRID:AB_2534095); goat anti-rat Alexa Fluor 488 (1:200, Thermo Fisher Scientific Cat# A-11006, RRID:AB_2534074); goat anti-rabbit Alexa Fluor 488 (1:200, Thermo Fisher Scientific Cat# A-11008, RRID:AB_143165); goat anti-mouse Alexa Fluor 488 (1:200, Thermo Fisher Scientific Cat# A32728, RRID:AB_2633277); donkey anti-goat Alexa Fluor 488 (1:200, Thermo Fisher Scientific Cat# A-11055, RRID:AB_2534102); horse anti-mouse IgG antibody, rat adsorbed (H + L) (1:200 Vector Scientific Cat# BA-2001, RRID:AB_2336180); goat anti-rabbit IgG antibody (H + L) (1:200 Vector Scientific Cat# BA-1000, RRID:AB_2313606); streptavidin, DyLight™ 594 (1:200 Vector Scientific Cat# SA-5594, RRID:AB_2336418); streptavidin, DyLight™ 488 (1:200 Vector Scientific Cat# SA-5488, RRID:AB_2336405). Two final washing steps were performed with 1 × TBS-T and 1 × TBS for 5 min prior to mounting with Shandon™ Immu-Mount (Thermo Fisher Scientific). For image acquisition and analysis, a Zeiss LSM 510 Confocal Microscope (Zeiss, RRID:SCR_018062) and the ZEN Digital Imaging software for Light Microscopy (Zeiss, RRID:SCR_013672) as well as ImageJ software (BioVoxxel, RRID:SCR_015825) were used, respectively.

### Corpus callosum dissection and RNA extraction

**Fig. 2:**
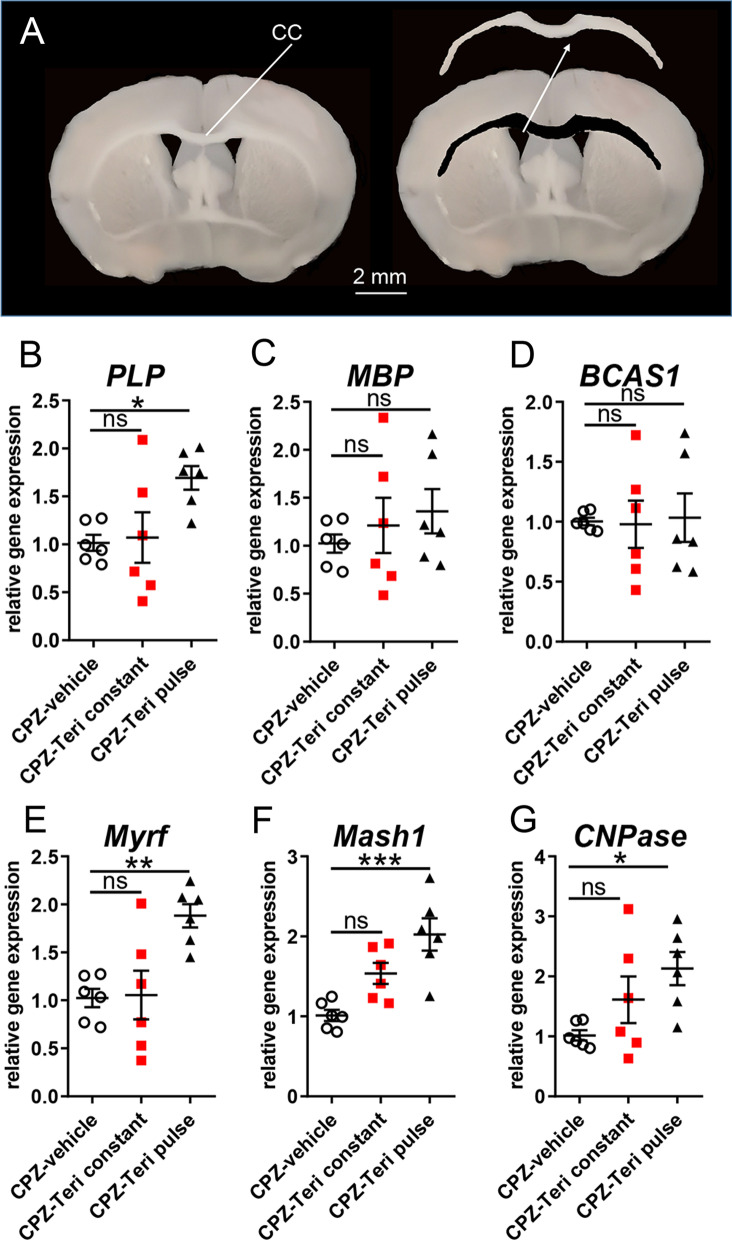
Gene expression responses upon teriflunomide stimulation. **A** Schematic presentation of corpus callosum tissue isolation for gene expression analysis (as shown here for the rostral CC, which was not analyzed in this study). **B**–**G** Quantitative RT–PCR of the dissected caudal corpus callosum 7d after cuprizone withdrawal (remyelination) indicated that pulsed teriflunomide application led to a significant upregulation of *PLP, Myrf*, *Mash1* and *CNPase* transcript levels, whereas *MBP* and *BCAS1* transcript levels were only mildly or not affected. *GAPDH* was used as reference gene. Number of animals per analysis n = 6. Data are shown as mean values (horizontal lines), while error bars represent standard error of the mean (SEM; vertical lines). Plots also show all individual data points. Statistical significance was calculated using one-way ANOVA with Tukey post-test (**B**–**G**). Data were considered statistically significant (95% confidence interval) at *p < 0.05, **p < 0.01, ***p < 0.001

Mice were deeply anesthetized using isoflurane inhalation and transcardially perfused with 20 ml PBS before decapitation and dissection of the brain. Serial 1 mm-thick coronal slices of the brain containing the CC were obtained by means of Brain Matrice Stainless Steel device coronal, 1 mm; Ted Pella, Cat# 15065). To minimize inclusion of tissue surrounding of the CC, the corpus callosum were isolated with direct visualization using a binocular dissecting microscope (Tritech Research Cat# S 217045664). Caudal samples of the CC were snap-frozen using liquid nitrogen and stored in RNAse free tubes (Fig. 2A).

RNA extraction from the corpus callosum samples was performed using TRIzol™ ( Thermo Fisher Scientific, Cat# 15065) reagent according to [25]. Frozen tissue sections were thawed on ice before 700 μL of TRIzol™ reagent were added. Polytron PT2100 cell shredder was used to break down cells. After an incubation of 5 min at RT 200 μL chloroform were added and the collection tube was shaken for 10–15 s (sec). Afterward, another incubation step of 5 min at RT followed, before samples were centrifuged for 15 min (4 °C, 12.000*g*). The RNA phase was carefully transferred to a new collection tube and 1 μL of glycogen was added for pellet visualization. In addition, 500 μL of isopropanol were added and the tubes were vortexed for about 5 s. Samples were then incubated for 15 min at RT and centrifuged for 10 min (4 °C, 12.000*g*). Isopropanol was carefully removed from the pellet. After washing with 1 mL 75% ethanol, tubes were vortexed and centrifuged for 5 min (4 °C, 12.000*g*). The supernatant was removed and samples were air-dried for 10–15 min until the rest of ethanol evaporated. Finally, RNA was eluted in 21.5 μL RNAse free water by incubating it on a heating shaker for 10 min (60 °C, 450 rpm). RNA concentration was determined using the NanoDrop ND-1000 spectral photometer (Thermo Fisher Scientific, RRID:SCR_016517) applying RNAse-free water as a blank. RNA was then stored at − 20 °C until cDNA synthesis was performed.

cDNA synthesis, and determination of gene expression levels by means of quantitative real-time RT-PCR were all performed as previously described [16]. Primer sequences were determined using PrimerExpress 2.0 software (Life Technologies) and tested for the generation of specific amplicons (sequences are available upon request). glyceraldehyde-3-phosphate dehydrogenase (GAPDH) and ornithine decarboxylase (ODC) were used as reference genes, and relative gene expression levels were determined according to the ΔΔCt method (Life Technologies). Each sample was measured in quadruplicate. Primer sequences: PLP_fwd: GGGCTCCCGGCCATATAA, PLP_rev: TCATCACCAGACAAGCAAAGAAA, MBP_fwd: ACAGAGACACGGGCATCCTT, MBP_rev: CACCCCTGTCACCGCTAAAG, BCAS1_fwd: CGCTGGGAAAGTTGTTTTGG, BCAS1_rev: TCTCCTCTGCACCTGTGGAAA, Myrf_fwd: GGACCCCAACTACCAATCCA, Myrf_rev: TGTCTTGACGTACTTGGGCT, Mash1_fwd: TCGTCCTCTCCGGAACTGAT, Mash1_rev: TAGCCGAAGCCGCTGAAGT, CNPase_fwd: CTGCCGCCGGGACAT, CNPase_rev: TCCCGCTCGTGGTTGGTAT, mfn1.1_fwd: GCAACCGAGAAGCTGCAGAT, mfn1.1_rev: CTGTACTTGGTGGCTGCAGTT T, mfn1.2_fwd: CAGTCCGGGCCAAGCA, mfn1.2_rev: GTGCAGGGAATCCATGATGAG, drp1_fwd: GCGCTGATCCCGGTCAT, drp1_rev: CCGCACCCACTGTGTTGA, pparg_fwd: CCCACCAACTTGGGAATCAG, pparg_rev: GGAATGGGAGTGGTCATCCA, ppargc1a_fwd: TGAAGAGCGCCGTGTGATT, ppargc1a_rev: TTCTGTCCGGGTTGTGTCA, ppargc1b_fwd: GGAAAAGGCCATCGGTGAA, ppargc1b _rev: GCTCATGTCACCGGAGAGATTT, pdpk1_fwd: AATGGTGAGGTCCCAGACTGA, pdpk1_rev: CCTGCTAACACCACTAGGAATGC, ldha_fwd: CTGTGTGGAGTGGTGTGAATGTC, ldha_rev: CAGCTGGGGGTTCAGAGACT.

### Electron microscopy

For electron microscopy, mice were transcardially perfused with 4% PFA and 2% glutaraldehyde in cacodylate buffer, then brains were dissected and post-fixed with 4% PFA and 2% glutaraldehyde overnight according to [26]. Appropriate regions of the corpus callosum were dissected, tissue sections were osmicated and processed for light and electron microscopy by dehydration and embedding in Spurr's medium. Ultrathin section (70 nm) were mounted to copper grids, counterstained with lead citrate, and investigated using a ProScan Slow Scan CCD camera mounted to a Leo 906 E electron microscope (Zeiss) and corresponding software iTEM (Soft Imaging System). Using morphological criteria, identified myelinated axons were counted and related to the total number of quantified axons.

### Analysis of mitochondrial dynamics

Changes in mitochondrial morphology were assessed from auto segmented images with an ImageJ macro “mitochondria analyzer” reporting several measures including mitochondrial count, length and mean form factor (FF; inverse “circularity” output value and defines a mitochondrial shape measure given by: perimeter^2^/(4πarea)) according to [27, 28]. The value 1 indicates round objects and this value increases with mitochondrial elongation, hence, allowing to distinguish two different morphologies from low (form factor 1) to high complex (form factor > 1) mitochondrial networks.

### Statistical analysis

Data are presented as mean ± standard error of the mean (SEM). Statistical analyses and graph design were performed using the GraphPad Prism 8.0.2 software (GraphPad Prism, San Diego, CA, RRID:SCR_002798). Shapiro–Wilk normality test was used to assess the absence of Gaussian distribution of all data sets. To determine statistical significance for normally distributed data sets one-way analysis of variance (ANOVA) with Turkey post-test for multiple comparisons was applied to compare three or more groups. For data sets not passing the Shapiro–Wilk normality test, Kruskal–Wallis test with Dunn’s post-test for multiple comparisons of three or more groups was applied. Statistical significance thresholds were set as follows: *p ≤ 0.05; **p ≤ 0.01; ***p ≤ 0.001 and ns = not significant. “*n*” represents the number of independent experiments. A priori sample size calculation for the in vivo experiments was performed using the G*Power 3.1.9.2 software [29] (test family: *t* tests; statistical test: means: Wilcoxon–Mann–Whitney test (two groups); tails: two; effect size d: 2.6; alpha error 0.05, power 0.95; allocation ratio N2/N1: 1; resulting maximal sample size: 6).

## Results

Since it was recently demonstrated that teriflunomide exerts direct effects on oligodendroglial cells in vitro and in vivo [16, 17] we further examined its potential for remyelination and restoration of subcellular structures.

### *Teriflunomide enhances oligodendroglial dynamics and remyelination *in vivo

To assess the extent of fostered remyelination by teriflunomide the cuprizone mediated mouse model of de- and remyelination was used [18]. Demyelination of the corpus callosum was initiated by feeding mice with 0.2% cuprizone diet for a total of 6 weeks. Based on our previous in vitro work demonstrating that teriflunomide signaling only induces its pro-oligodendroglial effects on OPCs when administered as short and early pulse [16] we applied two different schemes of teriflunomide administration in vivo (Fig. 1). During cuprizone challenge proliferation of parenchymal OPCs begins around week 3 with OPC densities peaking around weeks 4 and 5 [18, 30]. In an attempt to target these newly forming OPCs we administered the drug orally within drinking water as an early pulse (during the fourth week of cuprizone challenge for 4 consecutive days; “pulse”; Fig. 1) and also for a longer period during the final 12 days of cuprizone treatment (“constant”, Fig. 1).

**Fig. 3:**
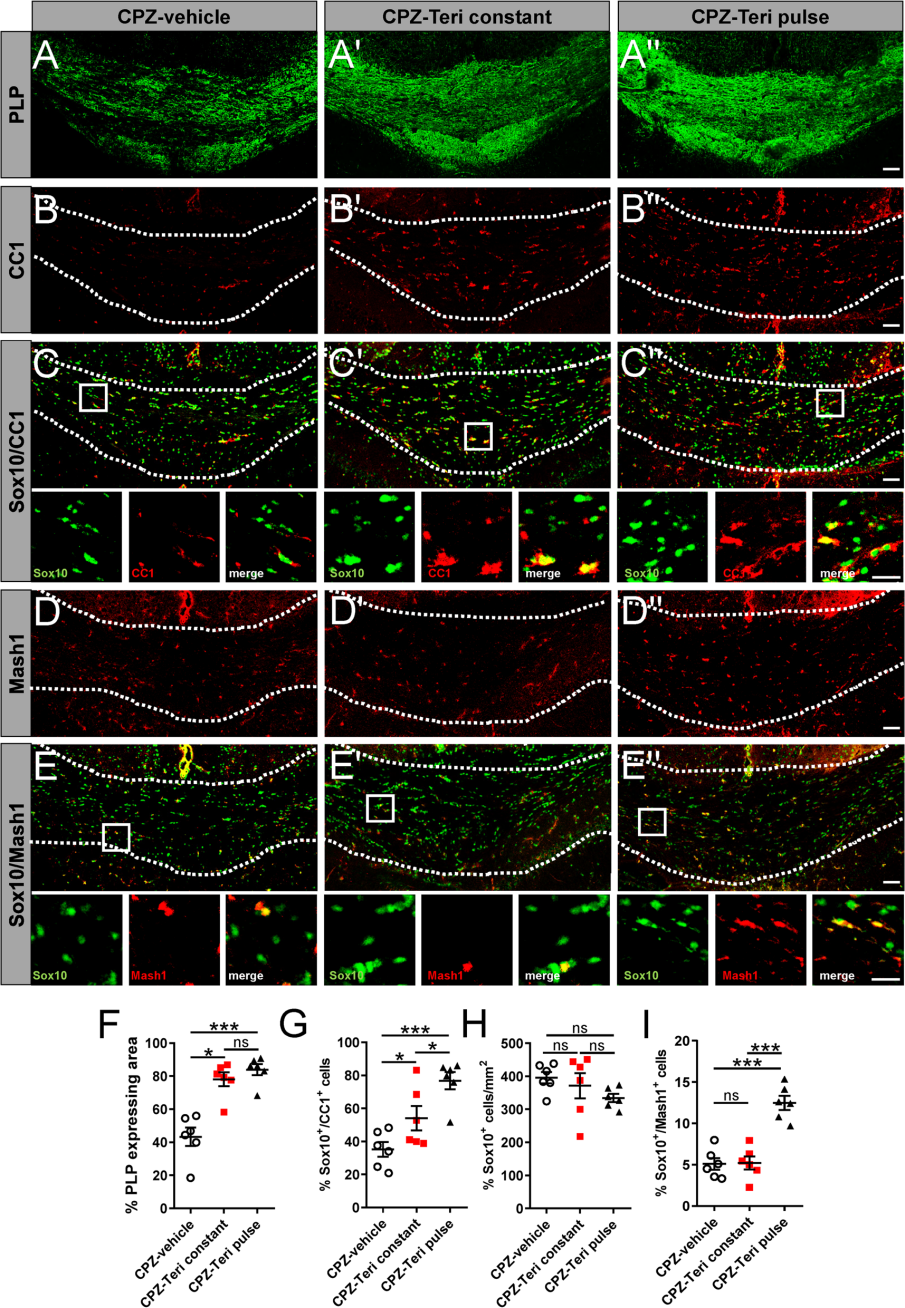
Teriflunomide administration positively affects remyelination-related oligodendroglial dynamics. **A**–**E**″ Representative images of oligodendroglial differentiation-associated protein markers in the caudal corpus callosum after 1 week of remyelination. **A**, **A″**, **F**) Degree of remyelination was determined as the percentage of PLP-positive area in the defined region of interest between CPZ-vehicle treated, CPZ with teriflunomide constant treatment (CPZ-Teri constant) and CPZ with teriflunomide pulse treatment (CPZ-Teri pulse) groups. The impact on oligodendroglial cell differentiation and maturation was assessed by the number/mm^2^ of Sox10-positive cells and the relative percentage of double positive cells (Sox10 + /CC1 + ; **B**, **C″**,**G**, **H**), (Sox10 + /Mash1 + ; **D**, **E″**, **I**) along the same area of the corpus callosum. Number of animals per analysis n = 6. Data are shown as mean values (horizontal lines), while error bars represent standard error of the mean (SEM; vertical lines). Plots also show all individual data points. Statistical significance was calculated using Kruskal–Wallis test with Dunn’s post-test (**F**, **G**, **H**) and Tukey’s range test following one-way ANOVA (**I**). Data were considered statistically significant (95% confidence interval) at *p < 0.05, **p < 0.01, ***p < 0.001. Scale bars: 50 µm, 20 µm

Analysis and quantification of the proteolipid–protein (PLP)-positive area in the caudal region of the corpus callosum revealed that both teriflunomide stimuli (constant and pulse) enhanced spontaneous remyelination as compared with vehicle-treated counterparts (Fig. 3A, A″, F). Furthermore, oligodendroglial cell dynamics reflected by the percentage of (Sox10-positive) oligodendroglial cells expressing the mature oligodendroglial marker adenomatous polyposis coli protein (APC) clone CC1 (CC1) supported this finding (Fig. 3B, C″, G). Total numbers of oligodendroglial lineage cells (as quantified using Sox10 expression) did not change significantly between the groups (Fig. 3H). Oligodendroglial cells expressing the essential transcriptional regulator Mash1 [23], previously described to be involved in teriflunomide’s pro-myelinating activity [16], were present in significantly increased numbers in treated animals as compared to vehicle control (Fig. 3D, E″, I).

### Evaluated gene expression responses upon teriflunomide stimulation

In line with the previous analysis, transcript levels of oligodendroglial differentiation-associated markers were determined by means of quantitative real-time PCR (qRT-PCR) of dissected CC tissues of the caudal region 1 week after remyelination (Fig. 2). Of note, only an early teriflunomide pulse (Fig. 1, early pulse) exerted an effect on myelin genes in that *2′,3′-cyclic-nucleotide 3′-phosphodiesterase (CNPase)* and *PLP* transcript levels were significantly upregulated (Fig. 2B, G). *MBP* transcript levels were elevated too, but reached no statistical significance and no regulation was observed for *breast carcinoma-amplified sequence 1 (BCAS1)* transcript levels (Fig. 2C, D). This response was further accompanied by an upregulation in the expression of key oligodendroglial transcription regulators (Fig. 2E, F) required for proper differentiation and myelin induction, such as *Mash1* [23] and *myelin regulatory factor (Myrf)* [31].

### *Teriflunomide enhances axonal remyelination *in vivo

**Fig. 4:**
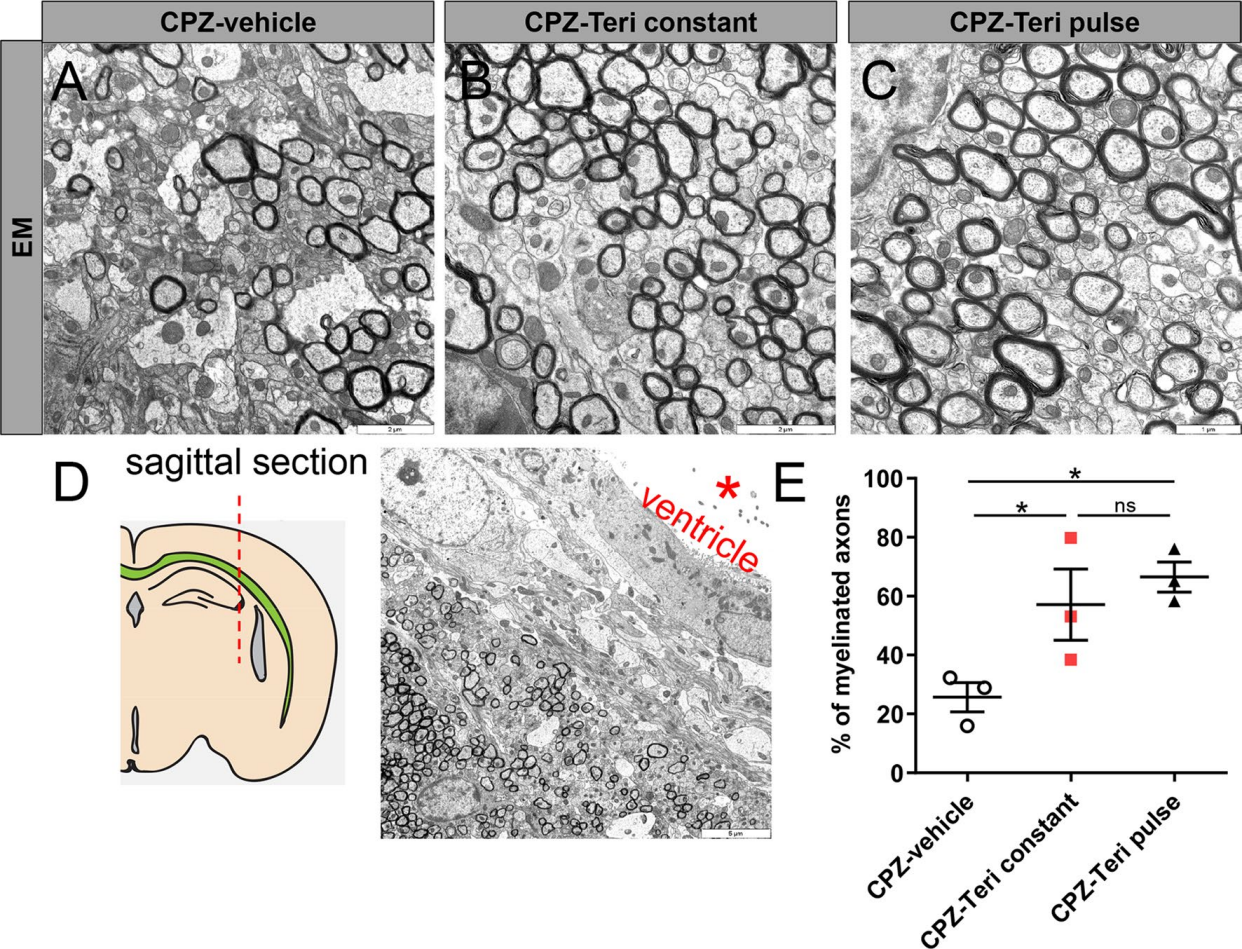
**A**–**C** Electron micrographs of sagittal corpus callosum (CC) sections from vehicle- and teriflunomide-treated mice showing myelinated axons (*n* = 3 per group). **A**, **B** Scale bars: 2 μM and **C** scale bar (1 µM). **D** Region of interest: sagittal section of the CC hitting the axons in cross section. Scale bar 5 µM. **E** Quantification of myelinated axons in corpus callosum revealed a significant increase upon both stimulation schemes (pulse and constant teriflunomide application). Data are shown as mean values (horizontal lines), while error bars represent standard error of the mean (SEM; vertical lines). Plots also show all individual data points. Significance was assessed using Tukey’s range test following one-way ANOVA (**E**). Data were considered statistically significant (95% confidence interval) at *p < 0.05, **p < 0.01, ***p < 0.001

To investigate whether the observed transcript and protein responses correlated with improved remyelination, the numbers of myelinated axons were quantified in electron micrographs from the caudal CC (Fig. 4A–E). One-week post-cuprizone application the corresponding quantification revealed a significant increase in the number of myelinated axons following both pulse and constant teriflunomide application as compared to control animals (Fig. 4E).

### Teriflunomide restores mitochondrial integrity in remyelinating oligodendroglial cells

**Fig. 5:**
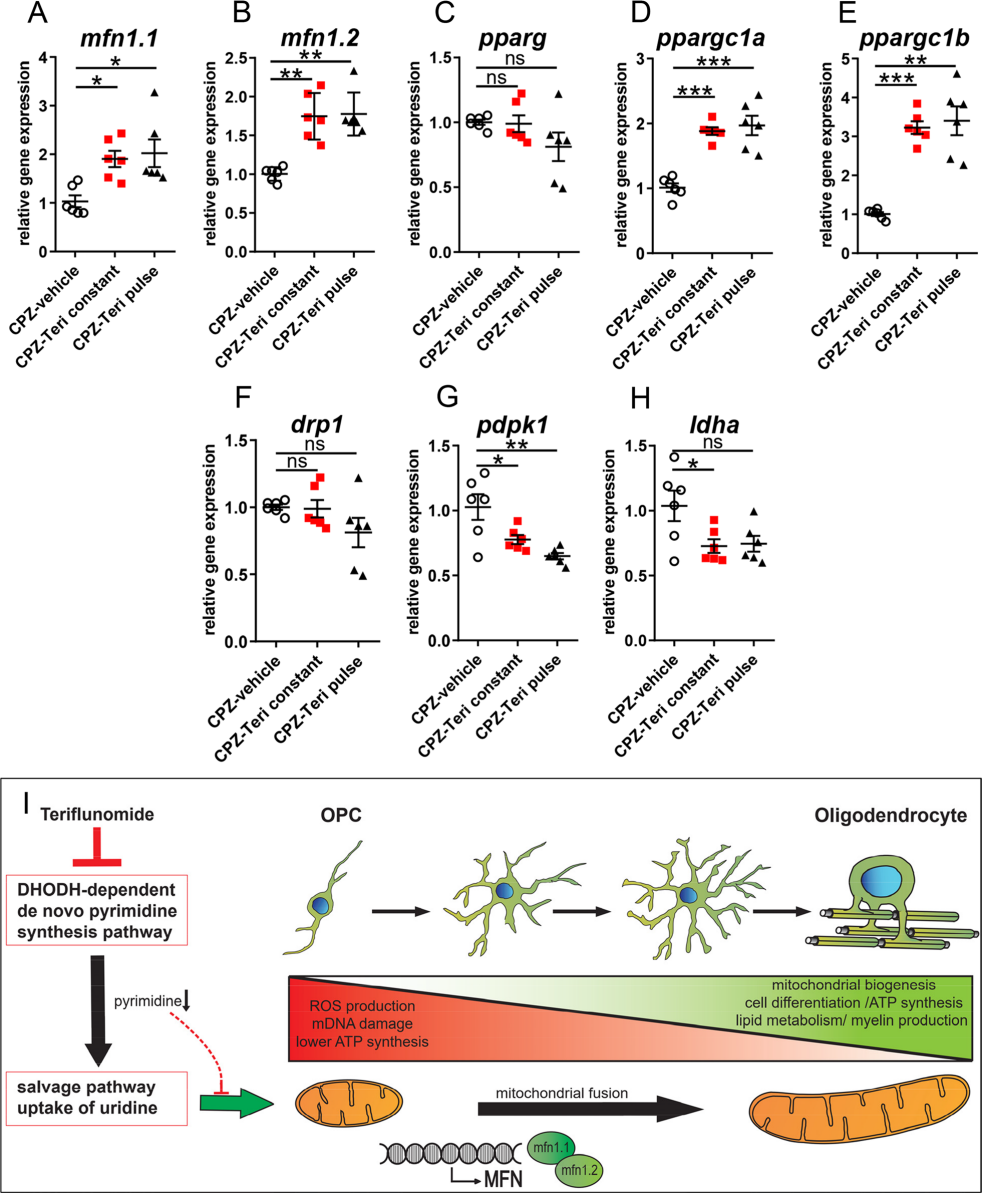
Mitochondrial gene expression responses upon teriflunomide application. **A**–**H** Quantitative RT-PCR of the dissected corpus callosum 7 days after cuprizone withdrawal (remyelination) indicated that both teriflunomide pulse and constant stimulation schemes led to a significant upregulation of genes catalyzing mitochondrial fusion such as *mfn1.1, mfn1.2, ppargc1a, ppargc1b* and to downregulation of genes responsible for glycolytic pathway, e.g., *pdpk1* and *ldha* transcript levels, whereas *drp1* (mitochondrial fission) and *pparg* were not affected. *GAPDH* was used as reference gene. **I** Illustration depicts mitochondrial dynamics involved in oligodendroglial differentiation, cell maturation and myelin sheath generation according to [49]. Fragmented and condense mitochondrial morphology is linked to ROS production, mitochondrial DNA damage and lower ATP synthesis. An elongated mitochondrial network hints to active mitochondrial biogenesis, ATP synthesis and lipid metabolism necessary for cell differentiation and generation of myelin. Teriflunomide induced the upregulation of mitofusins, and also mitochondrial elongation by depletion of the cellular pyrimidine pool secondary to DHODH inhibition. Number of animals per analysis *n* = 6. Data are shown as mean values (horizontal lines), while error bars represent standard error of the mean (SEM; vertical lines). Plots also show all individual data points. Statistical significance was calculated using Kruskal–Wallis test with Dunn’s post-test (**A**–**C**) and Tukey’s range test following one-way ANOVA (**D**–**H**). Data were considered statistically significant (95% confidence interval) at *p < 0.05, **p < 0.01, ***p < 0.001

All stages of oligodendroglial cell differentiation and especially the myelination process are characterized by an extensive mitochondrial demand related to biosynthesis and maintenance of myelin membranes [32]. We, therefore, examined whether mitochondrial homeostasis is affected in our model and in response to teriflunomide. We first investigated transcriptional changes in genes related to mitochondrial homeostasis (Fig. 5A–H). It was observed that both pulse and constant teriflunomide stimulation schemes led to significant upregulation of *peroxisome proliferator activated receptor gamma coactivator 1 alpha (ppargc1a)* and *peroxisome proliferator activated receptor gamma coactivator 1 beta (ppargc1b)* transcript levels which encode major regulators of mitochondrial biogenesis [33, 34]. Both teriflunomide treatment schemes also increased the expression of genes *mitofusin 1 and 2 (mfn1.1 and 1.2)* which are responsible for inducing mitochondrial fusion (elongation) and for preservation of mitochondrial DNA essential for mitochondrial function [35]. Accordingly, transcript levels of *dynamin-related protein 1 (drp1)* which is needed to induce the opposite process of mitochondrial fission (fragmentation) [36] were slightly (although not significantly) lowered upon teriflunomide application. These findings suggest that teriflunomide treatment promotes mitochondrial biogenesis and mitochondrial fusion as processes known for being associated with oxidative metabolism and more mature and differentiated cell types [37].

In agreement with an induction of mitochondrial metabolism, we observed that teriflunomide reduced the expression of the glycolysis gatekeeper *3-phosphoinositide-dependent protein kinase (pdpk1)* [38] as well as of *lactate dehydrogenase A (ldha),* the terminal enzyme in the glycolytic pathway responsible for pyruvate to lactate conversion [39]. Both genes have been associated with high proliferation rates [40] and their levels need to be decreased to enable proper cell differentiation [41].

**Fig. 6:**
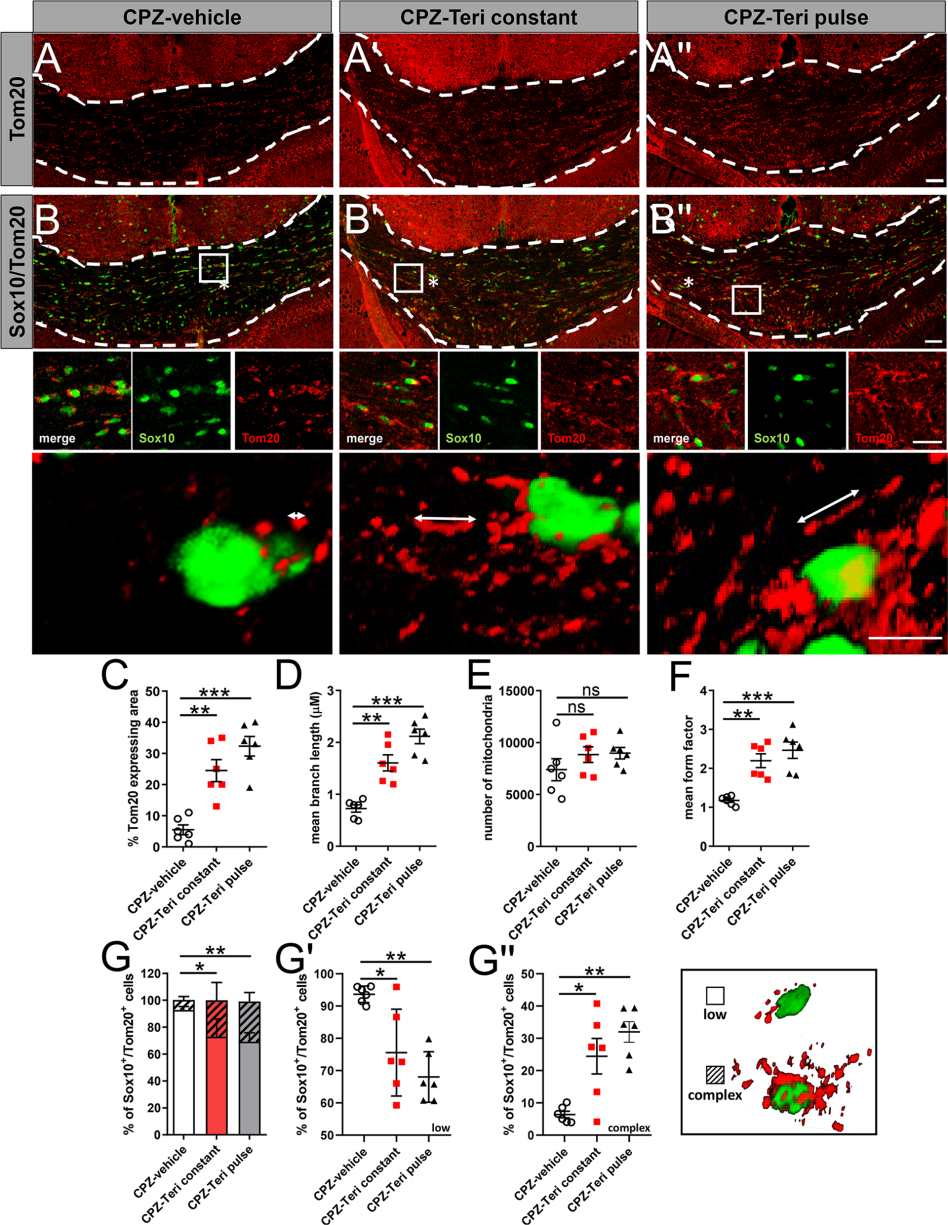
Teriflunomide mediated mitochondrial changes in oligodendroglial cells within the corpus callosum. **A**, **B″** Representative images of Sox10 and Tom20 double positive cells in the caudal corpus callosum after 1 week of remyelination. **A**, **B″**, **C** Extent of mitochondrial changes was revealed as the percentage of Tom20-expressing area in the defined region of interest (white borders). Further morphometric quantifications were assessed to determine mitochondrial, **D** length, **E** number and **F** mean form factor (FF). FF = 1 indicates round object, hence low length of mitochondrial networks within cells (form factor 1; low) and increases with elongation, to multiple mitochondria exhibiting elongated tubules (form factor > 1 complex). **G**–**G″** Moreover, the relative percentage of double positive cells (Sox10+ /Tom20+) containing few and less-elongated Tom20+ mitochondria categorized as “low” (blow up; asterix in B) where compared to Sox10-positive cells exhibiting Tom20+ mitochondria with elongated mitochondrial networks categorized as “complex” (blow ups; asterix in **B′**, **B″**. **G′** Individual data points related to the determination of double positive cells (Sox10 +/Tom20 +) categorized as “low” and (**G″**) individual data points for double positive cells (Sox10 +/Tom20 +) categorized as “complex”. Number of animals per analysis *n* = 6. Data are shown as mean values (horizontal lines), while error bars represent standard error of the mean (SEM; vertical lines). Plots also show all individual data points. Significance was assessed using Tukey’s range test following one-way ANOVA (**C**–**G**). Data were considered statistically significant (95% confidence interval) at *p < 0.05, **p < 0.01, ***p < 0.001. Scale bars: 50 µm, 20 µm, 10 µm

We next assessed the state of the mitochondrial network morphology in oligodendroglial cells of corpus callosum after 1 week of teriflunomide withdrawal by determining the expression of the mitochondrial outer membrane marker protein Tom20 (Fig. 6A, B″). Herein, teriflunomide treatment (pulse and constant) increased the overall Tom20 expression per area (Fig. 6A, B″, C). Furthermore, morphometric analysis according to [27, 28] indicated that both teriflunomide treatment schemes significantly increased mitochondrial branch length and form factor (FF) (Fig. 6D, F). The latter of which FF indicates an increase in mitochondrial elongation and interconnection, whereas no significant increase in the number of mitochondria could be observed (Fig. 6E). Furthermore, teriflunomide treatment decreased the fraction of Sox10-positive cells containing mitochondria with a fragmented shape (low), while elongated (complex) mitochondrial morphologies were enhanced (Fig. 6G, G″). Hence, these results indicate that teriflunomide promotes mitochondrial elongation which is in agreement with the observed upregulation of *mfn1.1/mfn1.2-* and downregulation *drp1* transcripts (Fig. 5A, B, F).

## Discussion

Currently used disease-modifying therapies (DMTs) mainly act on the immune system and are clinically used to reduce relapse occurrence and hence to slow down progression to persistent disability in MS patients. Teriflunomide is an approved first-line DMT for RMS and acts via blocking de novo pyrimidine synthesis thereby exerting a cytostatic effect on proliferating B- and T-cells [42]. However, teriflunomide withholds the potential to be employed as a regenerative compound operating beyond immunomodulation due to recent findings revealing teriflunomide to promote oligodendroglial cell homeostasis and myelination in vitro [16] and based on promyelinating effects in *Xenopus laevis* and in mouse spinal cords [17]. Consistent with these observations, we here demonstrate that teriflunomide fosters myelin repair within a toxin-based de-/remyelination model leading to accelerated generation of oligodendrocytes, restoration of myelin sheaths and amelioration of mitochondrial integrity. Of note, along the boosted oligodendroglial marker expression teriflunomide also promoted the expression of the differentiation associated transcription factors *Mash1* (Fig. 2F), a gene regulation which was also demonstrated previously in a stroke model within cells of the subventricular zone [43]. The initially described teriflunomide dependent pro-oligodendroglial effect via p73-signaling might, therefore, also be relevant in vivo [16].

Compared to previous in vitro studies [16] demonstrating promyelinating effects upon pulsed treatment only, it was of interest to examine that effects on spontaneous remyelination activities and gene expression responses as well as on mitochondrial dynamics occurred upon both pulsed and long-term teriflunomide application. Of note, the teriflunomide concentration used in this study [19, 20], resembles doses used in human patients and most importantly did not impact cell number/survival rates (see Fig. 3) as opposed to higher concentrations used in vitro [16]. Likewise, whereas in isolated OPCs, a prolonged teriflunomide application revealed to be counterproductive (as opposed to a maturation inducing pulsed stimulation), treatment of myelinating neuron/oligodendrocyte co-cultures appeared to be less sensitive in terms of timing [16]. Thus, this indicates that an increasingly complex environment supports a repair effect of this drug. And also, in the here presented in vivo paradigm long-term (constant) teriflunomide application did not negatively affect remyelination indicating that prolonged application as occurring in the context of the RMS therapy will not interfere with neuroregeneration. It is, therefore, tempting to speculate that the here described restoration of myelinated axons could in fact also contribute to the reduced disability progression in treated patients (TEMSO, ClinicalTrials.gov number NCT00134563; TOWER, ClinicalTrials.gov number NCT00751881) [44]*.* Nevertheless, it has to be taken into account that teriflunomide was applied orally thus limiting control over the consumed doses.

A growing number of studies suggest that lack of myelin repair and neurodegeneration are often attributed to a disturbance in mitochondrial homeostasis/metabolic function [45, 46]. Indeed, mitochondrial integrity has recently emerged as an essential modulator for neuronal and oligodendroglial cell differentiation [47, 48]. When compared with neurons and astrocytes, mitochondria length in oligodendroglia cell processes are between 0.8 and 2.3 µM and, therefore, approximately half as long as in astrocytes or neurons [49]. Indeed, in our analysis we measured mitochondrial lengths between 0.8 and 2.0 µM (Fig. 6D), hence, indicating oligodendroglial origin. An intact mitochondrial function is imperative for the increased energy demand during cell differentiation but also for lipid biosynthesis and the maintenance of myelin membranes [50, 51]. Assuring adenosine triphosphate (ATP) synthesis by means of oxidative phosphorylation (OXPHOS) [52, 53] is, therefore, critical for oligodendroglial cells to differentiate, mature and to build up myelin sheaths around axons [49]. Hence, these cells exhibit a high mitochondrial metabolism and contain increased densities of long, tubular (complex) mitochondria (Fig. 6D, F, G) [54]—a mitochondrial morphology that is thought to support high OXPHOS rates [55]. In the context of demyelination, mitochondria in both axons and OPCs are impaired, and lipid metabolism in OPCs within demyelinated lesions is disturbed [46]. Mitochondrial homeostasis and dynamics depend on two opposing processes, fission and fusion, the imbalance of which can result in cell damage, disturbed myelin repair and subsequent neurodegeneration [52, 56]. Fission, a mechanism promoted by drp1 (Fig. 5F), leads to shorter length, fewer cristae and fragmentation of mitochondria and is attributed to lower ATP rates in oligodendroglial cells [47]. Such mitochondria were mainly present within CPZ-vehicle treated animals following demyelination (Fig. 6B). Reduction of pyrimidine pools promoted by dihydroorotate dehydrogenase (DHODH) inhibition via teriflunomide not only triggers cell-cycle arrest [10, 16], but was also shown to modulate the expression of two highly conserved dynamin-related GTPases mfn1.1 and mfn1.2 (Fig. 5A, B) [57], thereby promoting mitochondrial fusion/elongation (Fig. 6B′, B″) [58–60]. Magalon and colleagues revealed that mitochondrial elongation is attributed to promoted oligodendrogenesis, as inhibition of mitochondrial fission/defragmentation fostered oligodendroglial cell maturation [61]. The underlying molecular mechanism of pyrimidine pool depletion resulting in altered mitofusin expression yet needs to be elucidated in future studies as it might be further explored therapeutically for sustained mitochondrial homeostasis and as an alternative approach to interfere with neurological dysfunction. Overall, our findings imply that teriflunomide promotes maturation and differentiation of OPCs also through a metabolic reprogramming that inhibits glycolysis and activates mitochondrial oxidative metabolism as well as mitochondrial biogenesis and fusion. Such modulated mitochondrial behavior might also be relevant for other neurological disorders, where mitochondrial fission is aberrant, such as, for example, Charcot–Marie–Tooth disease type 2A, a peripheral polyneuropathy caused by *mfn1.2* mutations [62]. Here, it could be of interest to examine the role of teriflunomide in suitable mouse models preceding clinical trials.

The applied toxin mediated demyelination model allows a precise and temporal analysis of neural cell responses to teriflunomide but lacks concomitant effects mediated to or via infiltrating immune cells. This apparent limitation, therefore, makes the translation of such regenerative effects to humans to be interpreted with caution. However, a previous study revealed teriflunomide to impair microglial activation in a mutant PLP mouse model [26], indicating that also resident immune cells are tamed further contributing to an overall beneficial effect. Accordingly, a recent clinical study indicated that teriflunomide treatment is associated with favorable outcomes regarding functional optic nerve recovery following optic neuritis in early multiple sclerosis [63].

Due to the fact that teriflunomide was applied within the cuprizone-mediated demyelination phase, one cannot fully exclude additional cytoprotective effects of teriflunomide to occur in vivo. Specific teriflunomide-related demyelination effects were not determined as animals were not killed at corresponding earlier timepoints. Based on our previous in vitro study [16] demonstrating a direct pro-oligodendroglial effect by teriflunomide, we specifically aimed at endpoint 1 week after cuprizone treatment to directly study oligodendroglial effects in vivo*.* This timepoint is in agreement with many other studies using this toxin-mediated demyelination model [64]. Based on the same literature [64] it is also clear that during the teriflunomide application period most demyelination already occurred. Hence, the observed positive effects on remyelination most likely result from an increased OPC differentiation potential, while minor additional effects can currently not be fully excluded.

Whether beyond oligodendroglia and microglia also other neural cells respond along this line needs to be shown. Nevertheless, teriflunomide was demonstrated to shift the astrocytic bioenergetic profile from oxidative metabolism to glycolysis and to attenuate TNFα-induced inflammatory responses [65]. Future investigations will, therefore, address to what degree this drug also ameliorates neurotoxic profiles of astrocytes as recently shown in a related mouse model upon medrysone treatment [66].

## Conclusions

The here presented study revealed that teriflunomide enhances endogenous myelin repair following cuprizone mediated demyelination. Hence, teriflunomide reflects an exciting translational candidate combining immunomodulatory and pro-regenerative properties. Moreover, this study revealed a novel link between de novo pyrimidine synthesis inhibition, stabilization of mitochondrial homeostasis and functional myelin repair. Thus, drugs affecting mitochondrial homeostasis could present further interesting approaches with clinical relevance for myelin repair and neuroregeneration.

## Data Availability

The data sets used and/or analysed during the current study are available from the corresponding author on reasonable request.

## Abbreviations

OPCs: Oligodendroglial precursor cells
NCS: Central nervous system
RMS: Relapsing MS
PMS: Progressive MS
MS: Multiple sclerosis
DHODH: Dihydroorotate dehydrogenase
CC: Corpus callosum
OLs: Oligodendrocytes
PFA: Paraformaldehyde
NGS: Normal goat serum
NHS: Normal horse serum
NDS: Normal donkey serum
Sox10: Sex determining region Y-Box 10
PLP: Proteolipid protein
Mash1: Mammalian achaete scute homolog-1
MBP: Myelin basic protein
BCAS1: Breast carcinoma amplified sequence 1
CNPase: 2′,3′-Cyclic-nucleotide 3′-phosphodiesterase
Myrf: Myelin regulatory factor
Mfn1.1: Mitofusin 1
Mfn1.2: Mitofusin 2
Drp1: Dynamin-related protein 1
Pparg: Peroxisome proliferator activated receptor gamma
Ppargc1a: Peroxisome proliferator activated receptor gamma coactivator 1 alpha
Ppargc1b: Peroxisome proliferator activated receptor gamma coactivator 1 beta
Ldha: Lactate dehydrogenase A
DMTs: Disease-modifying therapies
OXPHOS: Oxidative phosphorylation
ATP: Adenosine triphosphate
GAPDH: Glyceraldehyde 3-phosphate dehydrogenase
gr: Grams
g: G force
FF: Form factor
rpm: Revolutions per minute
min: Minutes
TBS: Tris-Buffered Saline
RT: Room temperature
sec: Seconds
h: Hours
d: Days
